# Early effects of ovariectomy on bone microstructure, bone turnover markers and mechanical properties in rats

**DOI:** 10.1186/s12891-022-05265-1

**Published:** 2022-04-02

**Authors:** Xingman Guo, Xiyue Yu, Qianqian Yao, Jian Qin

**Affiliations:** 1 Department of Radiology, The Second Affiliated Hospital of Shandong First Medical University, No.366 Taishan Street, Tai’an, 271000 Shandong China; 2 Xinjiang Medical University, Urumqi, 830054 Xinjiang Uygur Autonomous Region China

**Keywords:** Ovariectomized rats, Micro-CT, Bone microstructure, Bone metabolic indexes, Biomechanical properties

## Abstract

**Background:**

Fragility fracture is one of the most serious consequences of female aging, which can increase the risk of death. Therefore, paying attention to the pathogenesis of postmenopausal osteoporosis (PMOP) is very important for elderly women.

**Methods and materials:**

Forty 12-week-old female rats were divided into two groups including the ovariectomy (OVX) group and the control group. Four rats in each group were selected at 1, 4, 8, 12 and 16 weeks after operation. Vertebral bones and femurs were dissected completely for micro-Computed Tomography (micro-CT) scanning, biological modulus detection and histomorphological observation.

**Results:**

In OVX group, bone volume/total volume (BV/TV), bone trabecular connection density (Conn.D) and trabecular bone number (Tb.N) decreased significantly with time (*P <* 0.05). The elastic modulus of femur in OVX group was lower than that in control group, but there was no significant difference between them (*P >* 0.05). Over time, the tartrate resistant acid phosphatase (TRAP), osteocalcin (BGP), type I procollagen amino terminal propeptide (PINP) and type I collagen carboxy terminal peptide (CTX-I) in OVX group increased significantly (*P <* 0.05). The micrographs of the OVX group showed sparse loss of the trabecular interconnectivity and widening intertrabecular spaces with time.

**Conclusion:**

The bone loss patterns of vertebral body and femur were different in the early stage of estrogen deficiency. The bone turnover rate of OVX rats increased, however the changes of biomechanical properties weren’t obvious.

## Introduction

Mature bone requires continuous bone remodeling, which is the key mechanism to maintain normal bone mineral density (BMD). Therefore, bone formation is closely related to bone resorption and they are in a relatively balanced state in healthy individuals [1]. Postmenopausal osteoporosis (PMOP) is the most common primary osteoporosis in women. The main reason is that the sudden decline of estrogen level in the body leads to the imbalance of bone homeostasis in vivo, which accounts for the bone loss and fragility fracture eventually. Osteoporotic fragility fracture is one of the most serious consequences of female aging, because it increases the risk of death in elderly women [2, 3]. Therefore, paying attention to the pathogenesis of PMOP and preventing fragility fracture as soon as possible are very considerable for maintaining the bone health of elderly women and reducing the social burden of aging population.

In addition to BMD, changes in bone microstructure and bone strength should also be considered when predicting the risk of fragility fracture. Micro-Computed Tomography (micro-CT) can clearly describe the three-dimensional distribution and structural characteristics of bone. It also can measure accurately and monitor the changes of bone volume and bone microstructure [4–6]. Besides, serum bone turnover markers (BTMs) can timely reflect the metabolic level of bone formation and bone resorption, which is helpful to identify the high risk of fragility fracture [7, 8].

At present, many scholars have discussed the characteristics of PMOP from the perspective of bone mass, bone microstructure or bone metabolic indexes. However, there are few reports on the changes of bone microstructure, bone strength and blood biochemistry with time, especially in the early stage. In this study, the ovariectomized rat model was used to simulate the postmenopausal state of women. We aimed to explore the early manifestations of estrogen deficiency in rats’ axial bone and peripheral bone by micro-CT combined with BTMs and mechanical properties.

## Materials and methods

### Animals and experimental treatment

A total of 40 female Sprague–Dawley (SD) rats (220–250 g, 12 weeks) were purchased from the Beijing weitonglihua Experimental Animal Technology Co., Ltd. Then divided them into two groups with 20 rats per group. One group of rats underwent abdominal incision and bilateral ovariectomy (OVX). Subsequently, sutured the incision. The other group was the control group which is only performed abdominal incision without ovariectomy. Then the incision was sutured. The animals received ad libitum access to standard chow pellets and water throughout the experimental period.

At each time period of 1 week, 4 weeks, 8 weeks, 12 weeks and 16 weeks post-OVX surgery, four rats of each group weighted under anesthesia (3 ml of Urethane intraperitoneal injection, Shanghai Shanpu Chemical Co., Ltd). After that, they were killed by over anesthesia to get the L4 and L5 vertebral bones and bilateral femurs completely. Then stored them in 70% ethanol solution respectively. Animal usage and all procedures were in strict compliance with the guidelines of the National Institutes of Health and approved by the Shandong First Medical University Institutional Animal Care and Use Committee. This study is reported in accordance with ARRIVE guidelines.

### Micro-CT testing

**Fig. 1:**
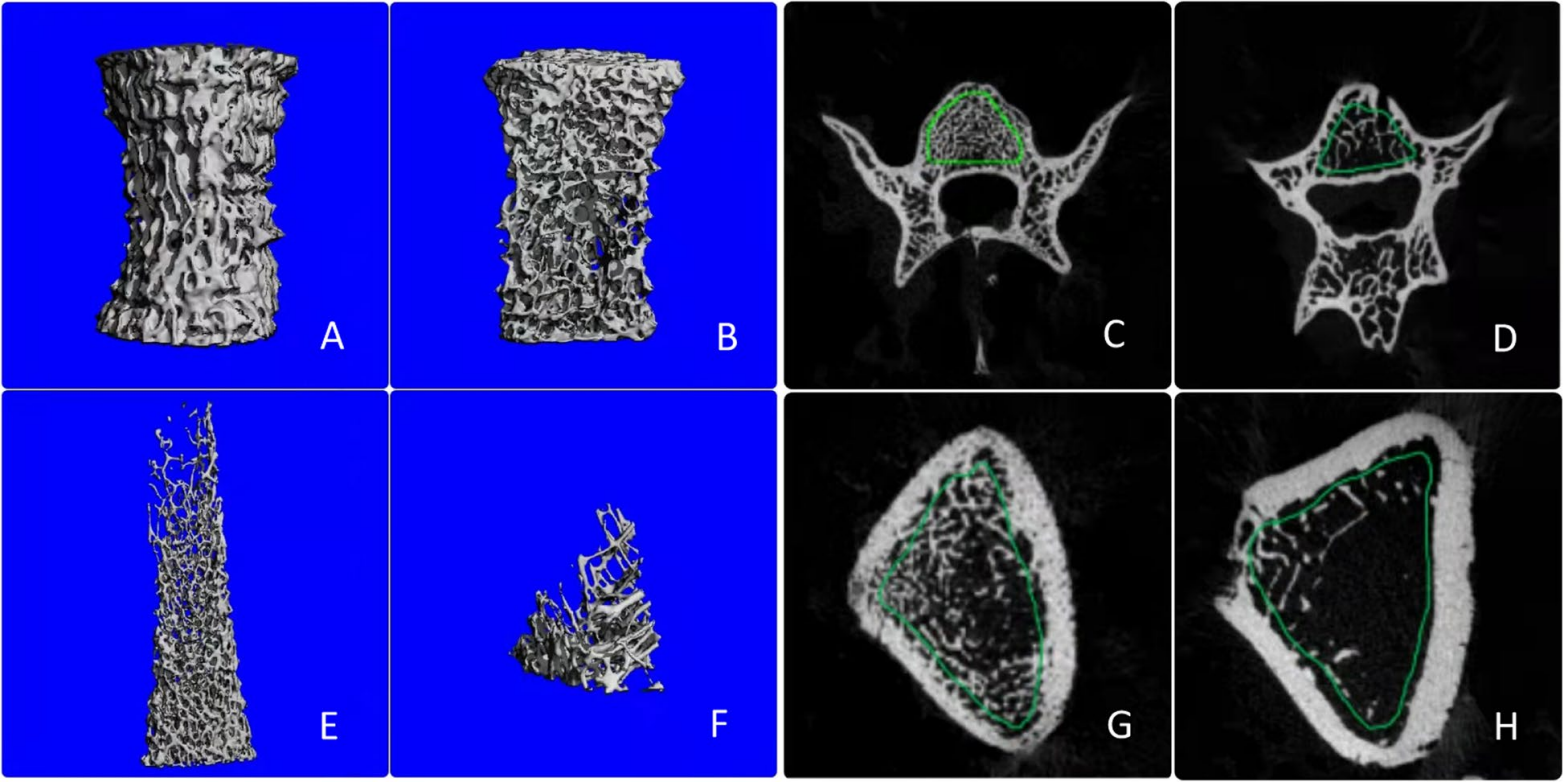
Three-dimensional reconstruction and cross-sectional images of L5 vertebral bone and left femoral shaft through micro-CT for OVX group rats. **A**-**D** Represents the vertebral bone. **E**-**H** Represents the femoral shaft. The first and third columns of pictures represent the 1th week and other columns of pictures represent the 16th week in OVX group rats. The region of interest is the volume of bone trabecular within the green line

Micro-CT of the harvested left femoral and L5 vertebral bones were acquired at 55 kVp, 145 μA for 221 ms, using Scanco μCT80 scanner (Scanco medical AG, Switzerland). All trabecular bone of the left femoral shaft and L5 vertebral bones were analyzed as the region of interest (ROI). See Fig. 1. The bone volume/total volume (BV/TV, unit %), bone trabecular connection density (Conn.D, unit 1/mm^3^), trabecular bone number (Tb.N, unit 1/mm), trabecular bone thickness (Tb.Th, unit mm) and trabecular bone separation (Tb.Sp, unit mm) were evaluated based on the micro-CT scan results.

### Biomechanical evaluation

The attached tissues of the right femur and L4 vertebral bones specimens of rats were removed. Then cut the vertebral facet joints, upper and lower endplates with serrated surgical scissors to obtain lumbar vertebra samples similar to a cylinder. The compressive stress modulus was tested by Instron testing machine. For right femur, the samples were placed on the center of the lower pressing plate with the patella facing upward. As for L4 vertebral bone, axially placed on the center of the lower pressing plate. The compression test was performed until the pressure load drops by 40% and then recorded the elastic modulus (E, unit MPa).

### Serum biochemical analysis

Five milliliter whole blood (each animal) was collected from the heart in terminal anesthesia through cardiac puncture. Centrifuged for 10 min at 3000 r.p.m. for serum separation. The tartrate resistant acid phosphatase (TRAP), type I collagen carboxy terminal peptide (CTX-I), osteocalcin (BGP), type I procollagen amino terminal propeptide (PINP), interleukin-6 (IL-6) and tumor necrosis factor-α (TNF-α) were determined by ELISA kits, using an autoanalyzer.

### Histomorphometric evaluations

The harvested bones including lumbar vertebra (L5) and left-femur were dissected to get free from soft tissues. Fixed them in 10% neutral buffered formalin solution. Then decalcified, dehydrated and embedded in paraffin. Subsequently, 5 μm thick sections were prepared and then stained with hematoxylin and eosin (HE). The histological sections used a microscope (20X) equipped with a camera to observe the morphological structure of trabecular bone.

### Statistical analysis

All statistical analyses were performed by SPSS software, Version 22.0. One-way ANOVA with subsequent LSD post-hoc test, independent t-test or rank sum test were used to compare the quantitative data between the groups and intra groups.

## Results

Over the entire study period, there was no significant difference and abnormality in the appearance of rats in OVX group and control group, including hair, paw, tail, etc.

### Rats body weight

The body weight of rats in both groups increased significantly with time (*P <* 0.05). At 4–16 weeks post-OVX surgery, the weight of rats in OVX group was significantly higher than that in control group (*P <* 0.05). The results are shown in Table 1.

**Table 1:** Weight (g) results of two groups in different periods

	Time	OVX Group (*n* = 4)	Control Group (*n* = 4)	*P*
Weight(g)	1 week	273.33 ± 15.27	279.67 ± 4.50	0.742
	4 weeks	384.50 ± 26.76	317.00 ± 25.91	<0.001
	8 weeks	409.50 ± 15.58	332.75 ± 8.65	<0.001
	12 weeks	446.75 ± 22.44	353.50 ± 35.40	<0.001
	16 weeks	472.00 ± 29.41	361.50 ± 24.40	<0.001
	*P*	<0.001	0.004	

Data are presented as mean ± SD. The body weight of rats in both groups increased significantly (*P <* 0.05)

### Micro-CT findings

In OVX group, BV/TV, Conn.D and Tb.N of vertebral bone and femur showed a time-course decreasing trend, whereas Tb.Sp demonstrated an increasing trend (*P* < 0.05). Besides, Tb.Th of vertebral bone and femur in OVX group and all indexes of vertebral bone and femur in control group did not show significant intra-group differences (*P* > 0.05). Specifically, BV/TV and Tb.Th differences of vertebral bone between the OVX group and control group occurred at the 4th week, while Conn. D, Tb.N and Tb.Sp occurred at the 12th week (*P* < 0.05). Moreover, BV/TV, Conn.D and Tb.N of femur were significantly lower than that in the control group, whereas Tb.Sp was significantly higher than that in the control group at 4–16 weeks post-OVX surgery (*P* < 0.05). Interestingly, Tb.Th of femur did not show significant inter-group differences at each time point (*P* > 0.05). The results are shown in Tables 2 and 3. Furthermore, three-dimensional reconstruction showed that the trabecular spaces followed a significant enlargement over time. The trabecular bone of cross-sectional imaging were partially broken and the number significantly reduced (Fig. 1).

**Table 2:** Results and comparison of micro-CT microstructure indexes of vertebral bodies between the two groups

	Time	OVX Group (*n* = 4)	Control Group (*n* = 4)	*P*
BV/TV (%)	1 week	0.41 ± 0.03	0.42 ± 0.08	0.628
	4 weeks	0.34 ± 0.04	0.46 ± 0.03	0.001
	8 weeks	0.34 ± 0.03	0.44 ± 0.05	0.005
	12 weeks	0.30 ± 0.03	0.45 ± 0.03	<0.001
	16 weeks	0.30 ± 0.02	0.47 ± 0.04	<0.001
	*P*	0.003	0.832	
Conn.D (1/mm^3^)	1 week	110.90 ± 18.05	115.99 ± 16.36	0.625
	4 weeks	90.63 ± 12.66	90.87 ± 22.21	0.982
	8 weeks	96.99 ± 9.13	84.43 ± 13.40	0.232
	12 weeks	73.71 ± 5.41	100.08 ± 15.51	0.016
	16 weeks	67.52 ± 8.99	97.93 ± 16.27	0.006
	*P*	0.001	0.155	
Tb.N (1/mm)	1 week	4.55 ± 0.38	4.69 ± 0.36	0.586
	4 weeks	4.23 ± 0.18	4.64 ± 0.61	0.117
	8 weeks	4.39 ± 0.27	4.78 ± 0.46	0.138
	12 weeks	3.88 ± 0.42	5.02 ± 0.19	<0.001
	16 weeks	3.69 ± 0.20	5.09 ± 0.29	<0.001
	*P*	0.008	0.470	
Tb.Th (mm)	1 week	0.08 ± 0.00	0.08 ± 0.01	0.452
	4 weeks	0.08 ± 0.00	0.09 ± 0.00	0.006
	8 weeks	0.07 ± 0.00	0.08 ± 0.00	0.019
	12 weeks	0.07 ± 0.00	0.08 ± 0.00	0.032
	16 weeks	0.07 ± 0.00	0.08 ± 0.00	0.017
	*P*	0.281	0.687	
Tb.Sp (mm)	1 week	0.20 ± 0.02	0.19 ± 0.02	0.826
	4 weeks	0.21 ± 0.01	0.19 ± 0.03	0.204
	8 weeks	0.20 ± 0.01	0.18 ± 0.01	0.251
	12 weeks	0.23 ± 0.03	0.17 ± 0.00	<0.001
	16 weeks	0.25 ± 0.01	0.17 ± 0.01	<0.001
	*P*	0.018	0.339

**Table 3:** Results and comparison of micro-CT microstructure indexes of femur between the two groups

	Time	OVX Group (*n* = 4)	Control Group (*n* = 4)	*P*
BV/TV (%)	1 week	0.12 ± 0.03	0.12 ± 0.05	0.738
	4 weeks	0.07 ± 0.02	0.16 ± 0.04	0.001
	8 weeks	0.06 ± 0.01	0.18 ± 0.03	<0.001
	12 weeks	0.05 ± 0.01	0.13 ± 0.05	0.001
	16 weeks	0.05 ± 0.01	0.13 ± 0.01	0.006
	*P*	0.002	0.206	
Conn. D (1/mm^3^)	1 week	49.36 ± 16.36	47.14 ± 24.78	0.842
	4 weeks	25.17 ± 8.45	76.88 ± 21.43	<0.001
	8 weeks	23.13 ± 7.29	63.82 ± 2.21	0.001
	12 weeks	14.29 ± 6.86	51.47 ± 29.70	0.002
	16 weeks	14.04 ± 1.88	48.01 ± 4.16	0.004
	*P*	0.001	0.216	
Tb.N (1/mm)	1 week	2.74 ± 0.39	2.36 ± 0.43	0.176
	4 weeks	1.28 ± 0.19	3.29 ± 0.51	<0.001
	8 weeks	0.82 ± 0.21	3.09 ± 0.21	<0.001
	12 weeks	0.96 ± 0.24	2.56 ± 0.78	<0.001
	16 weeks	1.09 ± 0.17	2.59 ± 0.22	<0.001
	*P*	<0.001	0.074	
Tb.Th (mm)	1 week	0.06 ± 0.00	0.07 ± 0.00	0.899
	4 weeks	0.06 ± 0.01	0.06 ± 0.00	0.083
	8 weeks	0.07 ± 0.00	0.07 ± 0.00	0.564
	12 weeks	0.07 ± 0.00	0.07 ± 0.00	0.831
	16 weeks	0.07 ± 0.00	0.06 ± 0.00	0.528
	*P*	0.313	0.312	
Tb.Sp (mm)	1 week	0.36 ± 0.06	0.44 ± 0.09	0.492
	4 weeks	0.81 ± 0.12	0.30 ± 0.05	<0.001
	8 weeks	1.31 ± 0.34	0.32 ± 0.03	<0.001
	12 weeks	1.10 ± 0.25	0.41 ± 0.12	<0.001
	16 weeks	0.97 ± 0.16	0.39 ± 0.03	<0.001
	*P*	<0.001	0.100	

Data are presented as mean ± SD

*BV/TV* The bone volume/total volume, *Conn.D* Bone trabecular connection density, *Tb.N* Trabecular bone number, *Tb.Th* Trabecular bone thickness, *Tb.Sp* Trabecular bone separation

### Biomechanical performance

In the temporal analysis for OVX rats, the elastic modulus of vertebral bone did not show significant intra-group differences (*P* > 0.05), however, the elastic modulus of femur increased significantly (*P* < 0.05). Compared to control group, the femur of OVX group showed inferior elastic modulus, whereas there was no significant difference between the two groups (*P* > 0.05). The results are shown in Table 4.

**Table 4:** Results and comparison of elastic modulus (MPa) of vertebral bone and femur between two groups

	Time	OVX Group (*n* = 4)	Control Group (*n* = 4)	*P*
Elastic modulus (MPa) of vertebral body	1 week	6.60 ± 0.77	5.78 ± 2.85	0.769
	4 weeks	9.88 ± 4.92	10.04 ± 1.86	0.954
	8 weeks	10.17 ± 1.97	10.06 ± 1.62	0.968
	12 weeks	11.11 ± 7.17	13.19 ± 3.52	0.454
	16 weeks	12.12 ± 5.59	12.78 ± 3.60	0.812
	*P*	0.557	0.015	
Elastic modulus (MPa) of femur	1 week	5.22 ± 0.89	8.47 ± 1.25	0.286
	4 weeks	14.72 ± 1.75	17.00 ± 6.04	0.416
	8 weeks	12.72 ± 1.79	14.72 ± 3.44	0.473
	12 weeks	14.93 ± 1.94	16.79 ± 2.22	0.507
	16 weeks	16.39 ± 4.52	19.30 ± 7.87	0.301
	*P*	<0.001	0.116	

Data are presented as mean ± SD

### Serum biochemical parameters

Over time, the levels of TRAP, CTX-I, BGP and PINP increased significantly in OVX group (*P* < 0.05). No statistically significant differences in the level of IL-6 and TNF-αin OVX group, although they were also increased with time (*P >* 0.05). Moreover, the level of TNF- α in the OVX group was significantly higher than the control group at 16 weeks after operation (*P* < 0.05). The results are shown in Table 5.

**Table 5:** Results and comparison of BTMs and inflammatory markers between the two groups

	Time	OVX Group (*n* = 4)	Control Group (*n* = 4)	*P*
TRAP	1 week	3.70 ± 0.53	3.37 ± 0.24	0.702
	4 weeks	2.99 ± 0.42	3.45 ± 0.67	0.491
	8 weeks	2.86 ± 1.56	3.62 ± 1.48	0.266
	12 weeks	1.87 ± 0.32	2.83 ± 1.02	0.158
	16 weeks	6.52 ± 0.49	6.89 ± 0.97	0.581
	*P*	<0.001	0.001	
CTX-I	1 week	0.53 ± 0.14	0.72 ± 0.26	0.351
	4 weeks	0.56 ± 0.14	0.50 ± 0.17	0.744
	8 weeks	0.39 ± 0.25	0.58 ± 0.36	0.243
	12 weeks	0.39 ± 0.08	0.45 ± 0.20	0.721
	16 weeks	0.95 ± 0.26	0.76 ± 0.23	0.237
	*P*	0.011	0.441	
BGP	1 week	12.99 ± 1.71	12.97 ± 2.24	0.996
	4 weeks	11.01 ± 0.50	10.36 ± 2.04	0.786
	8 weeks	6.20 ± 3.16	10.03 ± 3.93	0.119
	12 weeks	7.50 ± 1.56	10.76 ± 6.96	0.181
	16 weeks	12.19 ± 2.94	11.39 ± 2.89	0.738
	*P*	0.007	0.939	
PINP	1 week	11.90 ± 1.15	13.57 ± 5.12	0.554
	4 weeks	9.64 ± 1.30	8.22 ± 2.57	0.518
	8 weeks	6.11 ± 3.66	8.84 ± 5.02	0.218
	12 weeks	6.96 ± 1.84	8.89 ± 3.40	0.379
	16 weeks	14.88 ± 2.01	13.35 ± 2.92	0.488
	*P*	0.001	0.222	
IL-6	1 week	12.79 ± 1.08	13.66 ± 4.30	0.881
	4 weeks	11.93 ± 1.76	12.76 ± 1.27	0.853
	8 weeks	11.65 ± 1.30	10.86 ± 2.90	0.863
	12 weeks	13.21 ± 2.37	13.35 ± 3.57	0.977
	16 weeks	23.17 ± 17.06	14.35 ± 5.39	0.059
	*P*	0.284	0.734	
TNF-α	1 week	24.61 ± 3.57	28.02 ± 7.60	0.725
	4 weeks	25.72 ± 3.65	24.76 ± 2.83	0.898
	8 weeks	24.17 ± 2.47	22.61 ± 5.92	0.835
	12 weeks	25.42 ± 2.54	24.67 ± 5.63	0.920
	16 weeks	45.59 ± 28.06	28.41 ± 8.60	0.011
	*P*	0.172	0.704	

Data are presented as mean ± SD

*TRAP* The tartrate resistant acid phosphatase, *CTX-I* Type I collagen carboxy terminal peptide, *BGP* Osteocalcin, *PINP* Type I procollagen amino terminal propeptide, *IL-6* Interleukin-6, *TNF-α* Tumor necrosis factor-α

### Histopathological findings

**Fig. 2:**
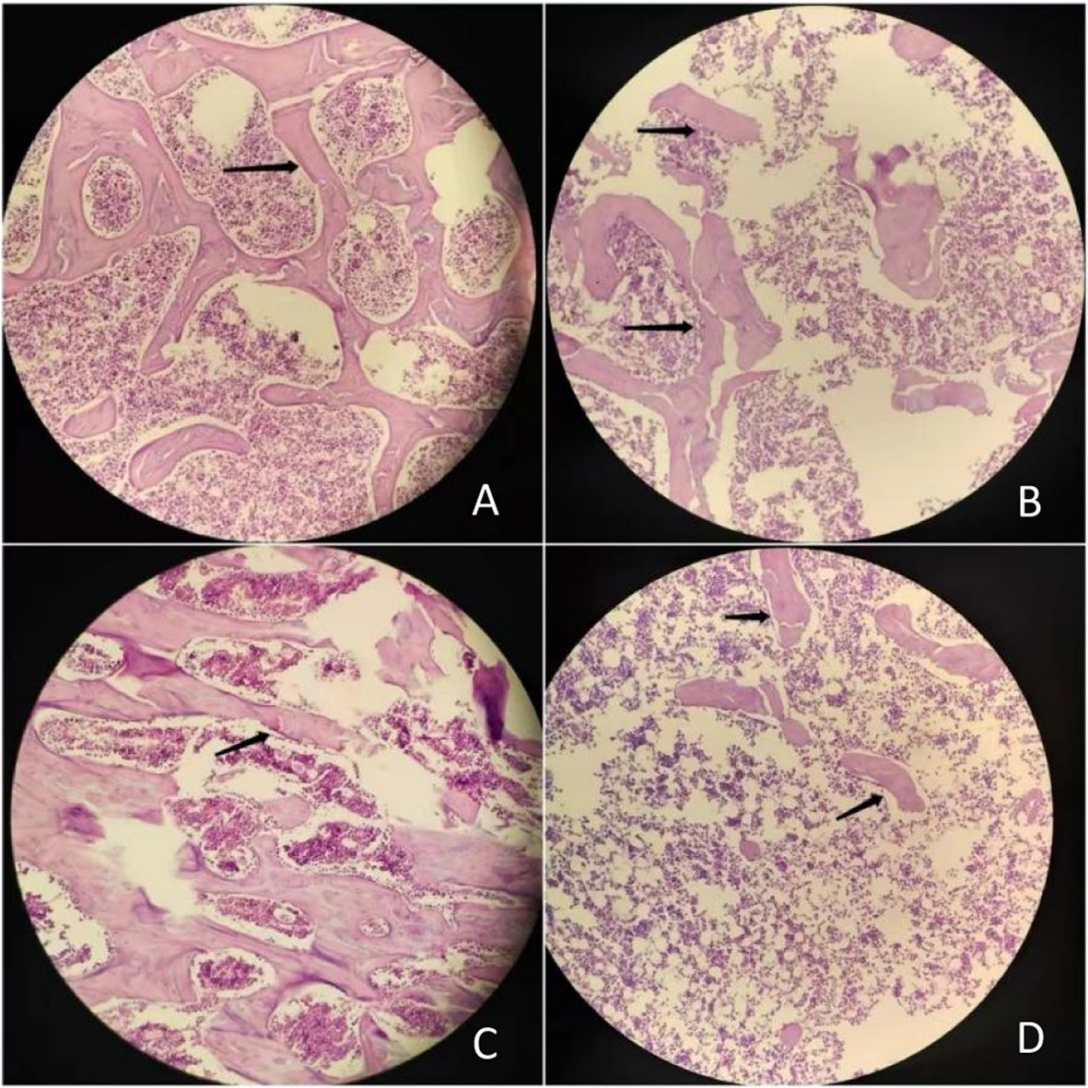
HE staining of OVX group. **A**-**D** The 1th week of L5 vertebral bone, the 16th week of L5 vertebral bone, the 1th week and the 16th week of left femoral head HE image (HE; magnification, 20X). The micrographs of OVX rats showed a typical osteopenia with loss of trabecular bone numbers and interconnectivity with time (arrow heads)

All HE-stained sections from two groups at different time were comparatively evaluated histologically (Fig. 2). Normal compactness of the vertebral body and femoral trabecular were found in the control rats. The lumbar vertebral and femoral head micrographs of the OVX group showed sparse loss of the trabecular interconnectivity and thinning of the trabecular, resulting in widened intertrabecular spaces over time.

## Discussion

With the prolongation of life span, the incidence rate of PMOP and fragility fracture is increasing year by year. According to the recent report showed that fragility fractures are related to up 50% of the disability rate and 20% of the mortality rate [9]. The medical expenses related to fragility fractures in China are expected to reach 163 billion RMB in 2050. Therefore, paying more attention to the pathogenesis of PMOP is very important for the life. It is now believed that the skeletal muscle, endocrine and digestive systems of rats are similar to those of humans in function and structure [10]. Moreover, ovariectomized rats have been proved to be one of the best models to simulate the clinical situation of postmenopausal human bone tissue. It is confirmed by tissue morphology that the osteoporosis model can be induced 3 months after ovariectomy [11].

In this study, the finding showed that the weight of OVX rats increased by 38% at 4 weeks after operation, and this result was consistent with the findings in other studies conducted on OVX rats [12]. Previous studies have demonstrated that 1–2 months old is adolescent mice and 3 months old is about 20–25 years old which reproductive development has matured [13]. Shi et al. in 2016 described that the L5 and L6 vertebral bones of 3–6 months old mice changed by only about 0.20 mm and the femoral length also increased by only 0.5–0.7 mm [14]. In other words, the bone growth of rats is almost completed after puberty so that the length of bone will not change much. We selected 12-week-old rats for ovariectomy in our study which could effectively abolish the significant effect of adolescent growth hormone on bone. Therefore, the estrogen was set as the single variable.

Bone is a composite biomaterial composed of 35–45% mineral crystals, 40% organic matrix and 15–25% water [15]. It has been reported that the bone loss of human postmenopausal axial bone is more obvious than that of peripheral bone [16]. After 16 weeks of operation, the vertebral trabecular parameters BV/TV and Conn.D decreased by 26.8 and 39.1% respectively in this study, while the femoral BV/TV and Conn.D decreased by 58.3 and 71.5% respectively. Interestingly, the degree of femoral bone loss was more obvious than that of vertebral body, which was in accordance with previous studies [17].

Tb.N, Tb.Th and Tb.Sp are main index to evaluate the spatial morphological structure of trabecular bone [6, 7]. It is reasonable to note that lumbar bone loss is mainly caused by bone trabecular thinning after OVX and the bone loss of tibia and femur are mainly caused by the disappearance of bone trabecular [18, 19]. Our study observed a similar phenomenon that vertebral body Tb.Sp (5.2%) of OVX rats was mainly caused by Tb.N declining (2.9%) rather than trabecular thinning (0.0%) at 1 week after operation. However, the thinning of bone trabecular led to Tb.Sp widening at 4–8 weeks after operation. Additionally, the bone loss of vertebral bone was related to Tb.N decline after 12–16 weeks of operation. As for the femoral Tb.Sp of OVX rats which was closer with Tb.N rather than Tb.Th at 1–16 weeks after operation. All values were expressed as a percentage of the absolute value of 1 minus OVX group / control group. It should be also noted that bone mass lose rapidly after estrogen deficiency and then reach the plateau stage which means a more balanced remodeling process. One theory considering that the homeostasis of bone reconstruction may be due to the compensatory thickening of bone trabecular after the 14 weeks of estrogen deficiency. However, the Tb.Th of vertebral body and femur of two groups in this study did not change notably with time and estrogen within 16 weeks after operation, which was similar to other findings [20].

In addition to bone microstructure, bone cortex plays a decisive role in bone biomechanical ability and fracture risk [20, 21]. In our study, the elastic modulus of vertebral body and femur of the two groups without decreased but increased. Consistent with those studies [22, 23], there were also no significant differences between the groups from 1 to 16 weeks after operation. It is now believed that the level of human BTMs will reach the lowest around the age of 35, and then increase rapidly and significantly during menopause [24]. Specifically, inflammatory cytokines also play an important role in bone remodeling and fracture risk [25, 26]. The International Osteoporosis Foundation and the International Federation of Clinical Chemistry recommend that all studies should at least include serum PINP (bone formation) and CTX-I (bone resorption) as references [27]. PMOP is characterized by a high bone turnover rate, which is opposite to the low bone turnover rate of senile osteoporosis [28]. Our study found that TRAP, CTX-I, BGP and PINP in the serum of OVX rats changed significantly with time, indicating that estrogen deficiency led to the acceleration of bone formation and bone resorption so that resulting the increase of bone turnover rate. In addition, we also observed the level of TNF-α of OVX group was significantly higher than that of the control group at 16 weeks after operation, which might be helpful to reflect the microenvironment state after estrogen deficiency as soon as possible.

Some limitations of this study need to be mentioned. Firstly, CTX-I is the product of type I collagen decomposition, which has a strong circadian rhythm and needs to be collected in the morning [29]. However, the blood collection was mainly at night in this study so that the final results might be affected to some extent. Secondly, although the bone changes after ovariectomy are consistent with the manifestations of osteoporosis, the level of estrogen in blood was not detected. Thirdly, due to the limitation of equipment, the stress of femur is not measured according to the “three-point bending” mode in most studies, but the results of elastic modulus should still have certain reference significance. In this study, the bone microstructure of rats before OVX was not evaluated, thus the related changes of bone within 1 week after OVX were not observed.

## Conclusion

In conclusion, the bone loss patterns of vertebral body and femur were different in the early stage of estrogen deficiency. The bone turnover rate of OVX rats increased, however the changes of biomechanical properties weren’t obvious.

## Data Availability

The datasets used and/or analysed during the current study available from the corresponding author on reasonable request.

## Abbreviations

BMD: Bone mineral density
PMOP: Postmenopausal osteoporosis
micro-CT: Micro-Computed Tomography
BTMs: Bone turnover markers
OVX: Ovariectomy
ROI: The region of interest
BV/TV: Bone volume/total volume
Conn.D: Bone trabecular connection density
Tb.N: Trabecular bone number
Tb.Th: Trabecular bone thickness
Tb.Sp: Trabecular bone separation
L: Lumbar vertebra
